# Social and economic variables related with Paraquat self-poisoning: an ecological study

**DOI:** 10.1186/s12889-020-08510-1

**Published:** 2020-03-27

**Authors:** Jefferson Antonio Buendía, Gabriel Jaime Restrepo Chavarriaga, Andres F. Zuluaga

**Affiliations:** 1 CIEMTO [drug and poison research and information center] at Integrated Laboratory of Specialized Medicine (LIME), Facultad de Medicina-IPS Universitaria, Universidad de Antioquia, Calle 64 #51-31, 050010 Medellin, Colombia; 2 Grupo de Investigación en Farmacología y Toxicología (INFARTO), Universidad de Antioquia, Medellín, Colombia

**Keywords:** Paraquat, Poisoning, Socioeconomic factors, Colombia

## Abstract

**Background:**

Paraquat self-poisonings constitute a significant contributor to the global burden of suicide. Our aim was to evaluate the relationship between social and economic variables with the incidence of self-poisoning with Paraquat in the northeast of Colombia.

**Methods:**

Records of 154 cases of self-poisoning with Paraquat and several socio-economic variables of six regions of northeast of Colombia were analyzed.

**Results:**

Most of the cases were mestizos, farmworkers, between 20 and 29 years, with intentional exposure using the oral route. Multivariate analyses revealed significant associations among the incidence of self-poisoning with PQ with the ecological factors such as poverty greater than 30% (IRR 15.9 IC95% 5.56–44.72), land Gini index < 0.7 (IRR 7.11 IC95% 3.58–14.12), private health insurance < 40% (IRR 3.39 IC95% 1.30–8.82) and planted area > 10% (IRR 2.47 IC95% 1.60–3.80).

**Conclusion:**

There is a relationship between ecological factors and, as such, this study opens the way to further developments in the field.

## Background

Suicide is still a significant public health problem worldwide with at least 800,000 deaths each year plus up to approximately 25 million non-lethal suicide attempts [1, 2]. Pesticide self-poisonings constitute a significant contributor to the global burden of suicide, accounting for at least one-in-seven fatal cases [3]. Despite the reduction in the number of pesticide suicides worldwide since 2006, the pesticides self-poisoning is still growing in Latin America accounting for approximately 10% of fatalities in this region [3]. The scenario could be worst, considering that the pesticide industry is forecast to grow annually by approximately 6% over the next few years in South America [4].

Suicidal behavior is a multifactorial event with diverse biological (e.g., sex, age, mental disorders, genetic loading, normal physical conditions, etc.) and sociocultural (e.g., unemployment, low income, lack of social support networks, ethnicity) determinants [5–8]. Most of these sociocultural determinants can be found in rural areas in developing nations [9]. However, studies have no focused on the relationship of these determinants with suicide attempts due to pesticides [9].

Among agrochemical products, paraquat is one of the most widely used for farming but also is commonly involved with self-poisoning [10–12]. After acute PQ poisoning, the fatalities rate exceeds 70% [9]. For example, according to the National Institute of Health in Colombia, pesticides resulted in 1231 deaths in the period between 2008 and 2015, with a worrying positive trend accentuated in the northeast of this country [13–17]. Here we reported a side product of the study entitled “Burden of paraquat poisoning in the department of Antioquia, Colombia” published elsewhere [18]. We consider that it is necessary to generate evidence to identify potentially modifiable social characteristics that allow reducing this problem. We aimed to evaluate the relationship between social and economic variables with the cases of self-poisoning with PQ in the northeast of Colombia in the period between 2010 and 2016.

## Methods

### Type of study

A mixed ecological study; ecological because it had the municipality as the unit of analysis, and mixed as it had, simultaneously, an exploratory character as well as a group comparison design [19].

### Ethics

No personally identifiable information was recorded all information obtained from health surveillance systems were kept confidential. Consent was not required because this was a study that used secondary sources of information already published. This study was approved by the Institutional Review Board of University of Antioquia (2015-4690). “under ‘Ethics Approval and Consent to Participate.

### Case definition

We included all consecutive patients with self-poisoning by paraquat, admitted in all hospital centers of the Department of Antioquia and reported by e-Formulary or in a physical paper to the Regional Epidemiological Surveillance System (SIVIGILA by its acronyms in Spanish) from January 1st, 2010 to the December 31st, 2016. This report is mandatory for all health care providers and made by personnel trained from each hospital. Antioquia is one of the 32 departments of Colombia, located in the central northwestern part of Colombia with a population of 6,613,118 [20]. This department can be subdivided into six regions: Magdalena Medio, Bajo Cauca, Urabá, Nordeste, Occidente, Norte, Oriente, Suroeste and Valle de Aburra.

### Population

The number of citizens in each region was taken from the official census established by the National Administrative Department for Statistics in Colombia [15, 21].

### Variables

The incidence rate was taken as the study’s dependent variable and was calculated for those regions corresponding to report to the SIVIGILA. The independent variables examined by region were the following: (i) amount of area planted with permanent crops, (ii) quality of life-index calculated by National Administrative Department for Statistics [21], which characterizes the living conditions of Colombians by related to housing (i.e, materials used in walls, floors and presence of public services), people (i.e., education, health, child care, labor force, expenses and income, etc.), and households (i.e., possession of assets and perception of the boss or spouse on living conditions in the home), (iii) net enrollment rate, that is the relation between the students enrolled that are in the appropriate age range for each educational level and the population in age of such school, (iv) the poverty level, indicates the percentage of people whose income is below the poverty line meaning that cannot satisfy their most basic needs, (v) drinking water coverage, (vi) private health insurance, (vii) heavy metal poisoning rate, (viii) Gini index distribution (20), that indicates the concentration of land ownership; and (ix) rate per 100,000 inhabitants of cases reported to SIVIGILA with psychological violence, physical violence, deprivation and negligence or suicide attempt [22].

These variables were dichotomized using the following cut-offs: (i) planted area of 10%, (b) the quality of life variable of 60% (c) the poverty level of 30%, (d) drinking water coverage of 40%, (e) enrolment rate of 70%, (f) the Gini index of land of 0.7, (g) deprivation and negligence of 8.70, (h) the heavy metal poisoning rate of 0.3 and (i) private health insurance of 40%. These cut-off points were used as suggested by the department of national economic planning [23].

### Database and analysis

Microsoft Excel 2016 was used to construct the database. Incidence rate ratios (IRR) were calculated using Poisson’s regression. We obtain at the incidence rate ratio by exponentiating the Poisson regression coefficient. The Poisson regression coefficients are the difference between the logs of expected counts to incidence rate ratio. Modeling and variable selection in the multivariate analysis were made following Greenland’s recommendations [24], We use The method of forward selection as follows: 1. Begin with no terms in the model. 2. Find the term that, when added to the model, achieves the largest value of R-squared. Enter this term into the model. 3. Continue adding terms until a preset limit on the maximum number of terms in the model is reached. All statistical analysis was done using Stata 11.0®.

## Results

In the period of the study, 154 cases of PQ poisoning were registered to SIVIGILA in Antioquia, of which 140 subjects were related to suicidal attempts. Most of the cases were presented in Uraba (*n* = 58), Oriente (*n* = 24), Norte (*n* = 20) and Bajo Cauca (*n* = 18). The case fatality in this study was 91 per 1000 patients (CI 95%: 46–136 per 1000 patients). Highest mortality rates were seen males, between 30 to 39 years old, with intentional ingestion of PQ. In general, the affected were mestizos and white, farmworkers, most of them between 20 and 29 years (*n* = 88, 57%), with intentional exposure per oral route, with a mean of 0.83 (IQR 0.002) days of length of delay between the onset/discovery/recognition of signs and symptoms of poisoning and a patient’s first visit to a health care provider.

**Fig. 1:**
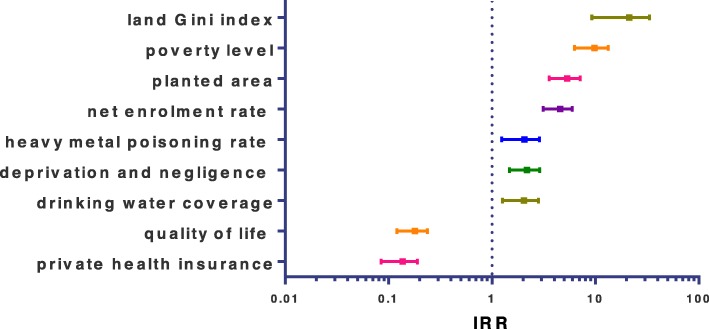
Graph showing the Incidence rate ratios (IRR) of self-poisoning by PQ

In the bivariate analysis, important associations with self-poisoning by PQ were found: planted area higher than 10% (IRR = 5.14; CI, 3.675–7.19), quality of life less than 60% (IRR = 0.17; CI, 0.125–0.241), poverty level higher than 30% (IRR = 9.34; CI, 6.474–13.471), drinking water coverage higher than 40% (IRR = 1.94; CI, 1.314–2.862), net enrolment rate less than 70% (IRR = 4.40; CI, 3.204–6.043), land Gini index less than 0.7 (IRR = 19.04; CI, 10.564–34.325), deprivation and negligence higher than 8.70 (IRR = 2.10; CI, 1.519–2.916), heavy metal poisoning rate higher than 0.3 (IRR = 1.95; CI, 1.299–2.915) and private health insurance less than 40% (IRR = 0.13; CI, 0.088–0.192) see Fig. 1. After modeling in the multivariate analysis, significant associations among the incidence of self-poisoning with PQ with independent variables were revelated such as poverty greater than 30%, land Gini index < 0.7, private health insurance < 40% and planted area > 10%, see Table 1.

**Table 1:** Results obtained in the multivariate model

Variable	IRR	95% Confidence Interval
planted area > 10%	2.47	1.60–3.80
quality of life < 60%	1.74	0.97–3.10
poverty level > 30%	15.90	5.65–44.72
land Gini index < 0.7	7.11	3.58–14.12
private health insurance < 40%	3.39	1.30–8.82

## Discussion

To the best of our knowledge, this is the first study relating economic inequalities in self-poisoning with PQ. This study demonstrates that poverty level, planted area, low equity of land distribution and lack of private health insurance were the most influential risk factors for self-poisoning with PQ in the northeast of Colombia.

These findings are highly consistent with previous reports in suicide for all causes, especially reported from developed countries [6–8, 25]. According to a systematic review, poverty, particularly in the form of worse economic status, diminished wealth, and unemployment is associated with suicidal ideations and behaviors [26]. In another study in 15 European countries, the suicide rate was negatively related to economic categorization; with affluent areas having the lowest rates and poor areas the highest of suicides [25]. Although the mechanisms of this relationship are unknown, income inequality can affect individuals by depression and feelings of hopelessness. Social and economic deprivations in more impoverished people, especially in rural areas, may make them disappointed and bring them closer to deciding to commit suicide [9] .

The lack of land distribution and planted area > 10% were independent risk factors associated with self-poisoning with PQ in our study. It is clear that regions with greater area planted have the higher risk of self-poisoning; especially if the farmers commonly stored more quantity of PQ within the household lacking education in good pesticide management [9, 27]. While in industrialized countries large-scale farming is practiced by a small number of landowners, in developing countries most of the farmers live in small areas of land and keep their supply of pesticides. These pesticides are generally are ease of availability and commonly stored within the household [28, 29]. Thus, the restriction on the use of pesticides is not enough to avoid this problem in developing countries; it must also be accompanied by government measures to improve the social conditions of farmers such as equality pi in the distribution of land. Indeed, areas with land Gini index < 0.7 have seven times more risk of cases of suicides by PQ. Also, areas without private health insurance – which in developing countries have better quality than public health system – have more risk of self-poisoning with PQ; this situation is due to the lack of legal health insurance by agricultural companies or small farmers. This is also reflected in the higher rates of informal work in these areas; which added to poverty, and inequality explain the high suicides rates in these regions. Intervention such as improved employment opportunities, welfare and mental health support services, as well as problem-solving skills development, may help farmers and reduce the burden of disease and suicides in poor areas [30, 31].

As this was an ecological study, it has potential limitations. Some other confounding variables could be no included in the present study. Nonetheless, when comparing our findings with those in previous descriptive and analytic studies, it was apparent that various of these studies also formerly associated poverty level or equity of land distribution with suicide for example. If information on birth and residence place had not been appropriately registered for reasons other than official ones, this study could have suffered from a selection bias. According to Susser [32], the exposure variables under consideration in this study were integral rather than to contextual. They were gathered from institutions with a long record in the analysis of ecological data and consequently, the risk of error in their classification should have been low. If the bias existed, it would have been non-differential, and the corresponding associations would have had tended to have a null value [33]. Although this study was carried out in the area with the highest incidence of PQ intoxication in the country, it is possible that the magnitude of these associations changes in areas with a lower incidence or other variables that were not included in the final model can be included or have interaction relationships not evidenced in this region of the country.

## Conclusion

The present study addresses the issue of ecological associations in self-poisoning with paraquat and, as such, opens the way to further developments in the field. The results presented here were based on a strict definition of case and used multivariate analysis to determine risks after adjusting possible confounding variables.

## Data Availability

All data generated or analyzed during this study are included in this published article**,** and are available and freely accessible in the report of the socioeconomic profile of Antioquia [23].

## Abbreviations

PQ: Paraquat
IRR: Incidence rate ratios
